# Fibrosis Microstructure Modulates Reentry in Non-ischemic Dilated Cardiomyopathy: Insights From Imaged Guided 2D Computational Modeling

**DOI:** 10.3389/fphys.2018.01832

**Published:** 2018-12-19

**Authors:** Gabriel Balaban, Brian P. Halliday, Caroline Mendonca Costa, Wenjia Bai, Bradley Porter, Christopher A. Rinaldi, Gernot Plank, Daniel Rueckert, Sanjay K. Prasad, Martin J. Bishop

**Affiliations:** ^1^School of Biomedical Engineering and Imaging Sciences, King's College London, London, United Kingdom; ^2^National Heart and Lung Institute, Imperial College London, London, United Kingdom; ^3^Biomedical Image Analysis Group, Department of Computing, Imperial College London, London, United Kingdom; ^4^Department of Cardiology, Guy's and St. Thomas Hospital Trust, London, United Kingdom; ^5^Institute of Biophysics, Medical University of Graz, Graz, Austria; ^6^Cardiovascular Research Centre and Cardiovascular Magnetic Resonance Unit, Royal Brompton Hospital, London, United Kingdom

**Keywords:** non-ischemic cardiomiopathy, computational modeling, late gadolinium enhanced magnetic resonance imaging, dilated cardiaomypothy, electrophysiology, reentry, arrhythmia (any), ventricular tachycardia (VT)

## Abstract

**Aims:** Patients who present with non-ischemic dilated cardiomyopathy (NIDCM) and enhancement on late gadolinium magnetic resonance imaging (LGE-CMR), are at high risk of sudden cardiac death (SCD). Further risk stratification of these patients based on LGE-CMR may be improved through better understanding of fibrosis microstructure. Our aim is to examine variations in fibrosis microstructure based on LGE imaging, and quantify the effect on reentry inducibility and mechanism. Furthermore, we examine the relationship between transmural activation time differences and reentry.

**Methods and Results:** 2D Computational models were created from a single short axis LGE-CMR image, with 401 variations in fibrosis type (interstitial, replacement) and density, as well as presence or absence of reduced conductivity (RC). Transmural activation times (TAT) were measured, as well as reentry incidence and mechanism. Reentries were inducible above specific density thresholds (0.8, 0.6 for interstitial, replacement fibrosis). RC reduced these thresholds (0.3, 0.4 for interstitial, replacement fibrosis) and increased reentry incidence (48 no RC vs. 133 with RC). Reentries were classified as rotor, micro-reentry, or macro-reentry and depended on fibrosis micro-structure. Differences in TAT at coupling intervals 210 and 500ms predicted reentry in the models (sensitivity 89%, specificity 93%). A sensitivity analysis of TAT and reentry incidence showed that these quantities were robust to small changes in the pacing location.

**Conclusion:** Computational models of fibrosis micro-structure underlying areas of LGE in NIDCM provide insight into the mechanisms and inducibility of reentry, and their dependence upon the type and density of fibrosis. Transmural activation times, measured at the central extent of the scar, can potentially differentiate microstructures which support reentry.

## 1. Introduction

Non-ischemic dilated cardiomyopathy (NIDCM) is characterized by enlarged ventricular cavity size, and impaired ventricular systolic function, which is not a consequence of myocardial ischaemia. Patients who present with NIDCM are known to be at high risk of sudden cardiac death (SCD), with an estimated 20% mortality rate over 5 years (Gulati et al., 2013). Late-gadolinium enhanced cardiovascular magnetic resonance studies (LGE-CMR) of NIDCM have highlighted that approximately one third of NIDCM patients have significant mid-wall fibrosis, corresponding to areas of enhanced image intensity (LGE), which are associated with an approximate 4- to 5-fold increased risk of sudden cardiac death (SCD) (Gulati et al., 2013). Despite this clear association, the specific physiological processes by which the structural remodeling associated with NIDCM underlies such increased arrhythmic burden remains poorly understood.

Further risk stratification in NIDCM based on LGE-CMR is therefore vital, yet is challenged by the lack of knowledge of both the underlying fibrosis micro-structure, as well as its implications for arrhythmogenic mechanisms. This is partially due to the limitations of current clinical scan resolutions, which are an order of magnitude larger than that required to resolve micro-structural fibrosis (Schelbert et al., 2010). Furthermore, detailed quantification of the absolute level of fibrosis in LGE images in non-ischemic patients is not possible, due to the lack of comparison between core scar and remote tissue, as is commonly used in assessing infarct fibrotic density in patients with ischemic cardiomyopathy (Glashan et al., 2018). This greatly confounds the identification of the potential fibrotic arrhythmogenic substrate in non-ischemic disease, and motivates a closer examination of how potential variations in fibrosis micro-structure underlying LGE may relate to differences in arrhythmia susceptibility and the mechanisms responsible for this.

In-light of the limitations of LGE in directly quantifying fibrosis density in non-ischemic disease, it may be possible to enhance risk stratification in NIDCM patients by combining LGE assessment along with functional electrical measurements, such as 12-lead ECG data or invasive electroanatomical mapping recordings (Betensky et al., 2013; Chia and Hsia, 2014), to better understand and assess the potential arrhythmogenic substrate evident on LGE-CMR. Facilitating such an approach first necessitates developing a greater understanding of how the fibrotic make-up of regions of mid-wall fibrosis may cause disruption to electrical wavefronts, under different conditions, which may be interpreted in functional electrical measurements.

Computational modeling provides a useful tool to better understand the mechanistic role of cardiac micro-structural remodeling in the initiation and maintenance of ventricular arrhythmias in the context of mid-wall fibrosis. Recently, detailed computational models have been applied to understand the arrhythmogenic interaction of electrical wavefronts and micro-fibrotic structures in a variety of pathological conditions such as modeling of atrial fibrillation (Roney et al., 2016; Vigmond et al., 2016) as well as micro-reentry (Alonso and Bär, 2013). In this study, we have developed the first computational model of fibrosis in NIDCM based on microstructural discontinuities. Based on a single LGE-CMR image, we explored possible variations in fibrosis micro-structure, and determined the consequences for reentry inducibility and reentry mechanism. Furthermore, we demonstrated the potential of transmural activation times (TAT) as a complementary source of data for the identification of arrhythmogenic substrate.

## 2. Methods

### 2.1. Image Acquisition and Processing

**Figure 1:**
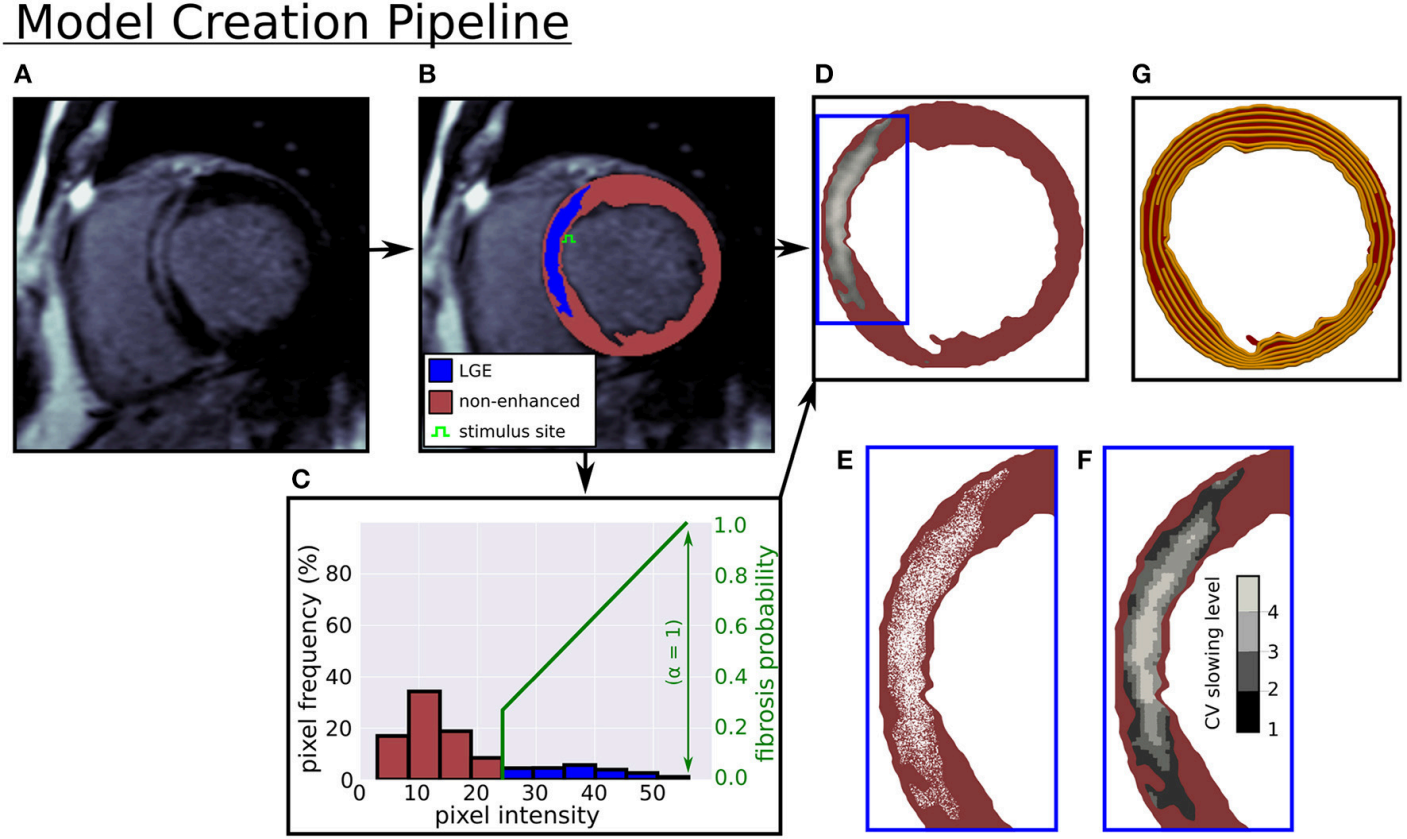
Computational model creation from an LGE-CMR image. **(A)** LGE-CMR image. **(B)** Image segmentation into enhanced and non-enhanced myocardium. **(C)** LV myocardial pixel intensity histogram and example intensity to fibrosis probability mapping. **(D)** Computational model with LGE intensities registered to image. **(E)** Zoomed in view of simulated fibrosis micro-structure. **(F)** Four conduction slowing zones in LGE. **(G)** Rule-based circumferential fibers.

An anonymized LGE-CMR image set of a patient presenting with NIDCM and LGE was acquired from the Royal Brompton Hospital, using a previously described protocol (Gulati et al., 2013), with approval of the UK National Research Ethics Committee [07/H0708/83, 09/H0504/104]. The patient gave written informed consent in accordance with the Declaration of Helsinki. The acquired images had a 0.66 mm^2^ in-plane resolution, and an 8 mm out of plane resolution. A single short axis (SA) image, with the largest LGE area (semi-automatic segmentation, 3 std > reference mean), was selected to build 2-D models (see Figure 1A).

### 2.2. Model Geometry

In Figure 1 the pipeline from LGE-CMR image to computational model is displayed. In this pipeline a triangular mesh (max 250 μm edge length) of the myocardium is made (CGAL), from smoothed (moving average filter) epi, endo, and LGE contours, with local myofiber orientations assigned by a 2-D version of a rule based method (Bayer et al., 2012). The generated fiber orientations were aligned primarily with the local circumferential direction (see Figure 1G).

### 2.3. Fibrosis Microstructures

Interstitial and replacement fibrosis were modeled by modifying mesh edges and triangles, respectively, with a single parameter α controlling the density of fibrosis in LGE. The parameter α was selected from the range 0−1 in steps of 0.1, which yielded models with varying levels of fibrosis. Additionally, each model's level of fibrosis was related to the normalized image intensity

(1) $$\begin{array}{l} I^{*}=\frac{I-I_{ref}}{I_{max}-I_{ref}}, \end{array}$$

with *I*, *I*_*max*_, *I*_*ref*_ denoting the local, maximum, and mean non-LGE reference image intensities respectively, so that higher intensity areas contained more fibrosis. A fibrosis probability was assigned to each mesh triangle or edge, giving a probabilistic distribution of replacement or interstitial fibrosis at each level of α. Concrete realizations of these fibrosis distributions were created by generating a random number for each edge or triangle, and assigning it to be fibrotic if the random number was less than the local fibrosis probability.

For replacement fibrosis, the probability of a mesh triangle being fibrotic was

(2) $$\begin{array}{l} p_{replacement}=\alpha I^{*}, \end{array}$$

where α is the global fibrosis density. Fibrotic triangles were removed from the mesh, making them electrically inert and non-conducting.

Interstitial fibrosis was represented by a network of random fibrotic clefts (Costa et al., 2014), implemented by doubling mesh vertices across fibrotic edges. Each doubled vertex was assigned to a different neighboring mesh element along the edge, thereby creating a local no-flux boundary condition aligned with the mesh edge. The probability of an edge being fibrotic was

(3) $$\begin{array}{l} p_{interstitial}=\alpha \cos^{4}\left(\theta \right)I^{*}, \end{array}$$

where θ is the angle between the triangle edge and the local myocardial fiber direction. The cos^4^(θ) term in the equation greatly decreases the likelihood of fibrosis not aligned with the myocardial fiber direction. This ensures a highly anisotropic fibrosis distribution, which is what we would expect in the case of interstitial fibrosis.

**Figure 2:**
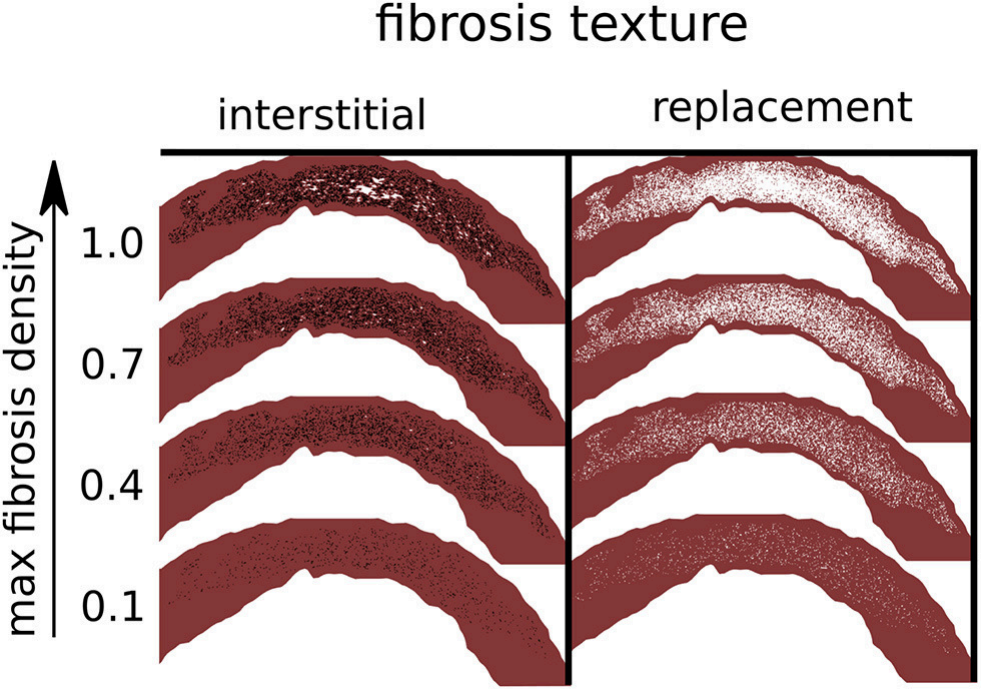
Simulated fibrosis micro-structures with varying type and density. Black lines are separating edges through which current cannot cross, whereas white areas are electrically inactive and non-conducting.

Model tissues within circuits of interstitial fibrosis were removed, since they were electrically isolated. Example models with both interstitial and replacement fibrosis at various densities are shown in Figure 2.

### 2.4. Conductivities and Conduction Velocities

Conductivity values in non-enhanced areas were tuned to match conduction velocities (CV) from NIDCM (Anderson et al., 1993), 84 and 23 cm/s in the fiber and transverse directions, respectively.

Both normal conduction (NC) and reduced conduction (RC) were considered in LGE. In the case of NC the same conductivity values were used as in non-enhanced areas. In the case of RC, model LGE areas were assigned one of four reduced CVs based on image intensity. Regions in the intensity range 0–25 and 25–50% above threshold had transverse conductivity (CVT) reduced by 25 and 50%, respectively, with normal fiber direction conductivity (CVF). Finally, regions in the intensity ranges 50–75 and 75–100% above threshold had CVF reduced by 25 and 50%, respectively, and CVT reduced by 50%. These four reduced sets of CV values are consistent with (Anderson et al., 1993), which reported a correlation between fibrosis level and CV slowing, as well as a tendency for preserved longitudinal CV with milder levels of fibrosis.

### 2.5. Dataset of Fibrosis Microstructures

We constructed a set of model fibrosis micro-structures, consisting of variations in type (replacement, interstitial), conductivity (normal, reduced) and 10 levels of maximum density α (0.1–1.0). Ten random realizations of fibrosis were made for each combination of conductivity, fibrosis type, and fibrosis density, along with a control model with normal conductivity and no fibrosis. This gave a total of 401 models (Figure 2).

### 2.6. Electrophysiology Simulation

Electrical activity was simulated by the standard monodomain formulation, with ionic currents represented by the Ten Tusscher and Panfilov (2006) model of the human ventricular action potential, integrated with step size 20 μs. Due to the lack of experimental evidence for ionic remodeling in NIDCM, the standard ventricular ionic properties for the Ten Tusscher and Panfilov (2006) model were used. Both monodomain solver and cell model are implemented in the Cardiac Arrhythmia Research Package (CARP)(Vigmond et al., 2003).

Models were paced primarily from a site on the LV septal endocardium, approximately halfway down the extent of the LGE (see Figure 1B). Two additional endocardial sites were used to perform a sensitivity analysis. These sites were located at three quarters and at the limit of the LGE extent respectively (Figure **7**). Stimuli had a square shape with 500 μm edge length, strength 500 μA/cm2, and duration 2 ms. Activation times were recorded at the first time that the transmembrane voltage *v*_*m*_ crossed 0 and $\frac{dv_{m}}{dt}>0$.

### 2.7. Pseudo-Electrograms

Pseudo-electrograms were calculated by estimating the extracellular potential (Bishop and Plank, 2011)

(4) $$\begin{array}{l} \phi_{e}=\frac{1}{4\pi \sigma_{b}}\int_{\Omega }\frac{\nabla \cdot \left(\sigma_{i}\nabla v_{m}\right)}{\left||r|\right|}d\Omega \end{array}$$

where Ω is the cardiac domain, σ_*i*_, σ_*b*_ = 1.0*S*/*m* the tissue conductivity tensors for the intracellular domain and outside of the heart respectively, and ||*r*|| the distance to the electrogram measurement point, which was 4 cm anterior to the computational geometry.

### 2.8. Stimulation Protocols

Activation patterns and transmural activation times were generated with a fixed stimulation protocol, consisting of 5 beats with coupling intervals (CIs) 500, 340, 250, 210 ms.

Reentry inducibility was tested with a dynamic algorithm, with up to six stimuli with variable CI. The algorithm consists of the components: reentry detection, coupling interval selection, and stimulus capture detection.

#### 2.8.1. Reentry Detection

For each stimulus 600 ms of electrical activity were simulated, and electrical activations were recorded. Reentry was detected if any activations were present after 170 ms in a region of interest (ROI). For all experiments the ROI was defined by a circle with radius 3 mm, centered at the stimulus site, and excluded fibrotic areas.

#### 2.8.2. Coupling Interval Selection

The coupling intervals (CI) between stimuli were determined algorithmically for every stimulus after the first. First the CI of 200 ms was attempted, and if the resulting stimulus generated a new wave, then this CI was kept. Otherwise the effective refractory period (ERP) at the stimulus site was determined, and the CI was set to ERP + 1 ms. This ensured that electrical stimuli were delivered rapidly enough to destabilize the model, and yet slowly enough that each stimulus generated a distinct wave. An effective lower bound of 200 ms was placed on the CI, as pacing rates faster than this can generate reentries under non-pathological conditions (Cao et al., 1999). ERP was calculated to within 1 ms by a binary search when it was required, with an initial bracket of [200, 400 ms].

#### 2.8.3. Stimulus Capture Detection

Knowledge of whether a stimulus generated a new activation wave was required for the coupling interval selection. This was accomplished by tracking a wave of activations for 20 ms after a stimulus was applied, with a stimulus determined as capturing if activations could be tracked up to 20 ms. Initially, all activations inside the reentry detection ROI were found 1 ms after stimulus delivery. For each subsequent millisecond new activations were located inside a set of tracking ROI, which consisted of model tissue that was within the distance that an activation wave could travel in 1 ms, assuming the maximum CV of 84 cm/s.

### 2.9. Reentry Classification

**Figure 3:**
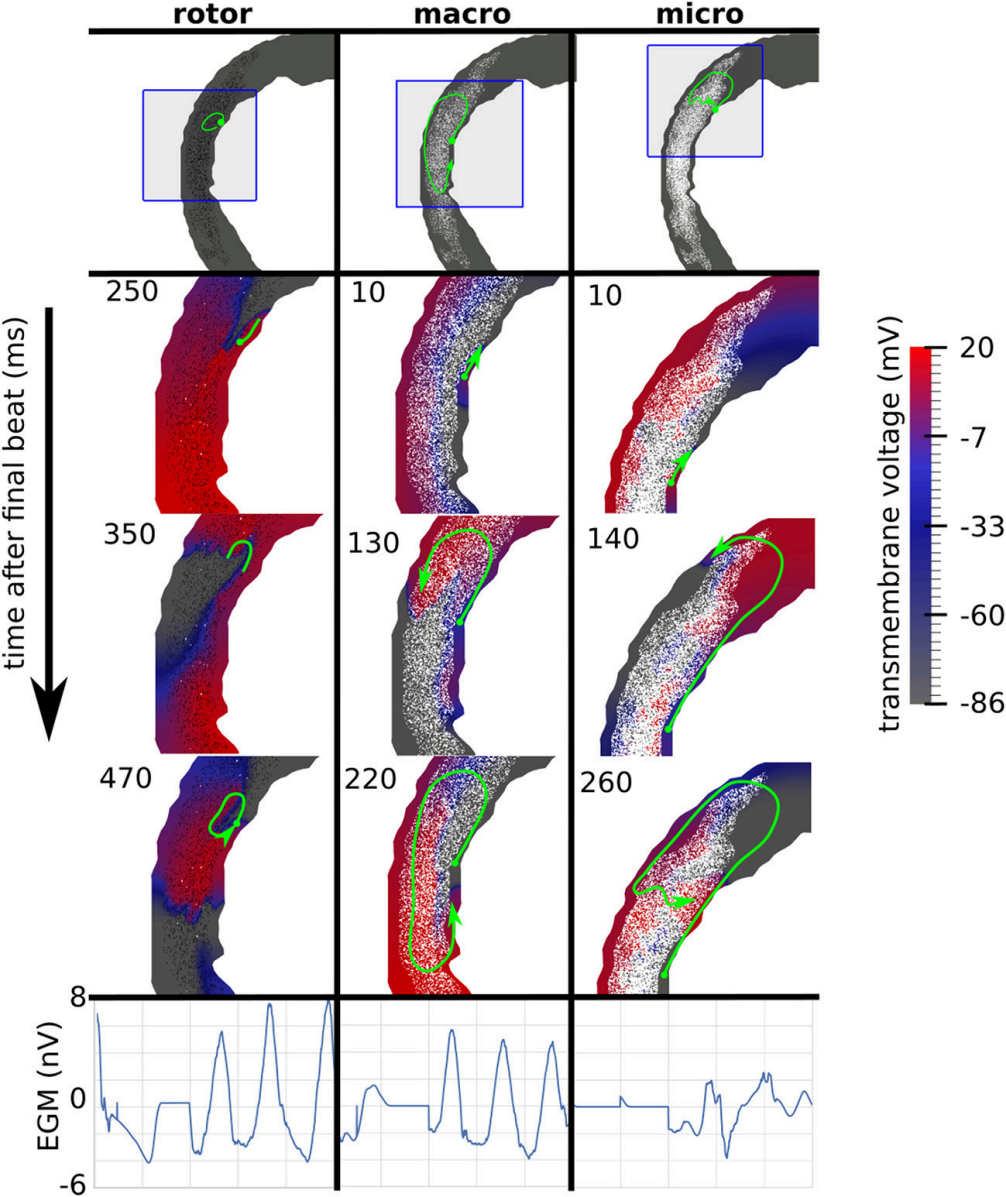
Examples of three canonical reentry mechanisms. **(Top)** Paths of reentrant circuits in green, with blue boxes indicating zoomed-in areas used for the voltage maps below. **(Middle)** Transmembrane voltage maps, with time (ms) after the final stimulus displayed in the top left corner. **(Bottom)** Pseudo-electrograms measured 4 cm anterior to the geometry showing 1s of electrical activity.

Reentries were classified by visual inspection of videos of the simulated transmembrane potential. A reentry was classified as a rotor if an organizing center was present, consisting of nearly simultaneous activation and repolarization, as micro-reentry if a narrow conducting channel was involved, and as macro-reentry if activation followed a large circuit. When multiple reentry types were present in a simulation, the reentry type of the earliest circuit was used. Examples of the three reentry types are shown in Figure 3, and in the Supplemental Videos.

## 3. Results

### 3.1. Fibrosis Texture, Density, and Conductivity Modulate Reentry Inducibility

**Figure 4:**
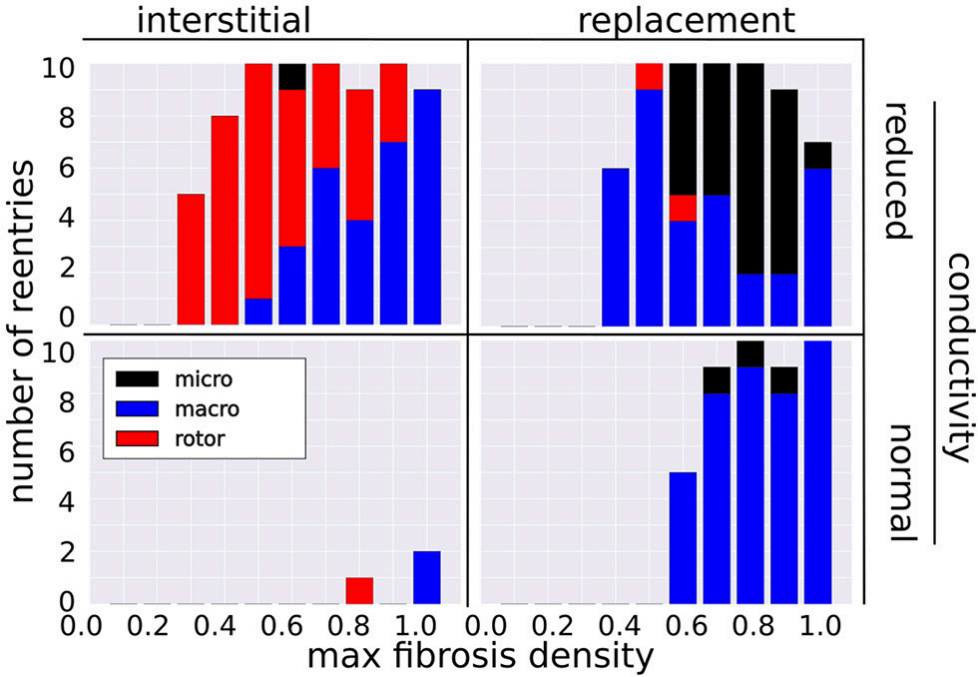
Incidence of reentry mechanisms by fibrosis texture, density, and with normal and reduced conductivity in the LGE.

Figure 4 shows the number of reentries per fibrosis texture, density and conductivity. In total 181 reentries were induced: 107 were due to the replacement texture whereas 74 were due to the interstitial texture. The presence of reduced conduction in LGE was associated with a greater reentry incidence (48 reentries for NC versus 133 with RC), with most of the increase being due to interstitial fibrosis (3 reentries NC vs. 71 with RC). For all fibrosis types there was a minimum level of fibrosis density necessary for reentry. This threshold was lower with reduced conduction, being RC 0.2, 0.4, NC 0.8, 0.6 for interstitial and replacement textures, respectively.

### 3.2. Reentry Mechanisms Vary With Fibrosis Type and Density

The incidence of each reentry mechanism is shown in Figure 4. Rotor reentries mostly occurred with interstitial texture, RC, and lower density, whereas macro-reentries occurred with replacement texture, and for higher densities of interstitial texture with RC. Micro-reentries were prevalent in the maximum density range 0.6–0.9 with RC and replacement texture. The pseudo-electrograms for the example canonical reentries (Figure 3) showed differing morphologies, with the rotor and macro-reentry having a signal indicative of monomorphic VT, whereas the micro-reentry showed a more fractionated signal, due to wavefront disorganization. We note the relatively small signal amplitude of the pseudo-electrograms, which we attribute to the small fraction of the total LV mass being represented in our 2-D models.

### 3.3. Transmural Activation Time Increases With Fibrosis Density and Higher Pacing Rate

**Figure 5:**
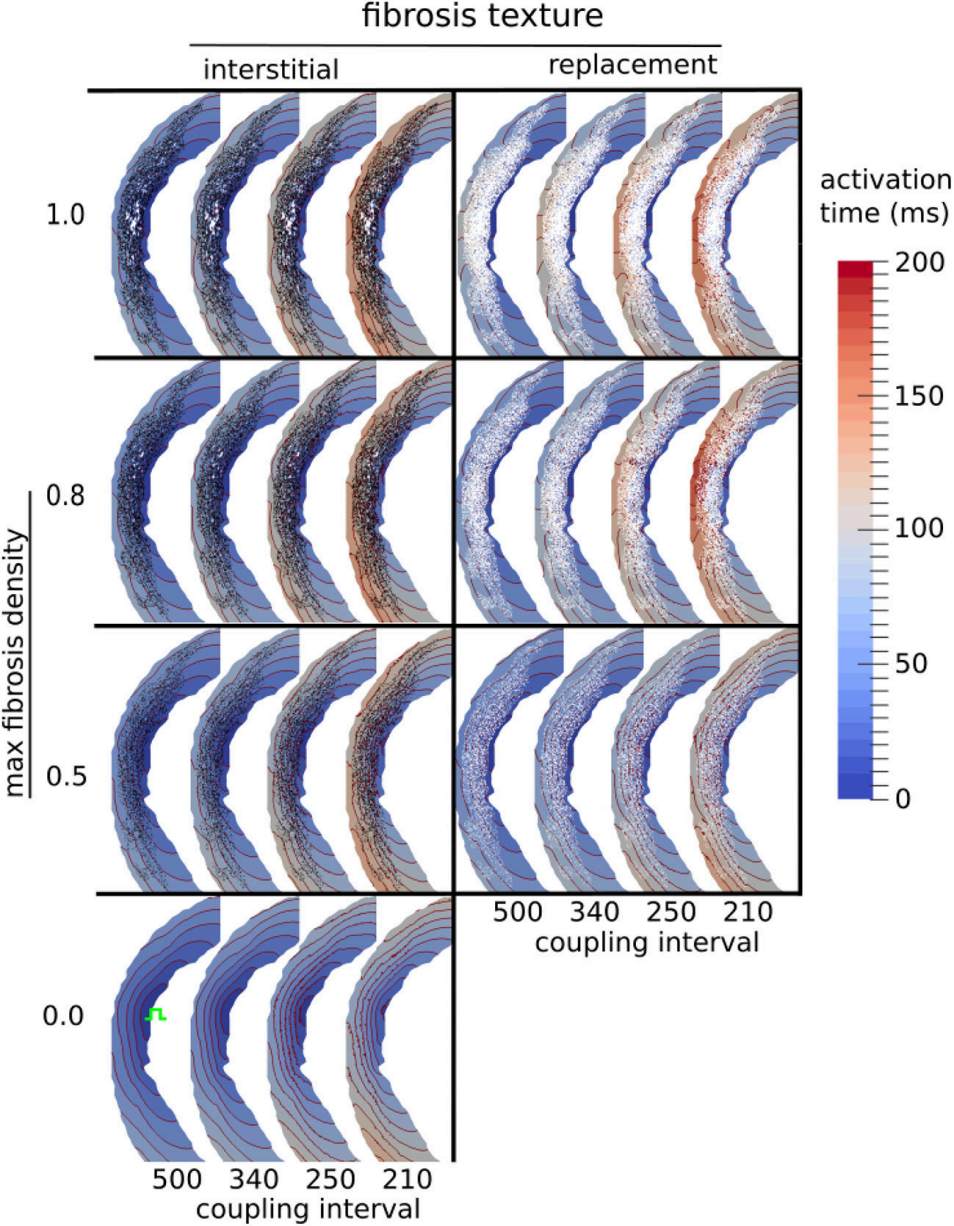
Activation maps around the LGE for three types of fibrosis micro-structure. Red lines are isochrones spaced at 10 ms intervals. The stimulus site is shown in green in the fibrosis-free control model (bottom row).

**Figure 6:**
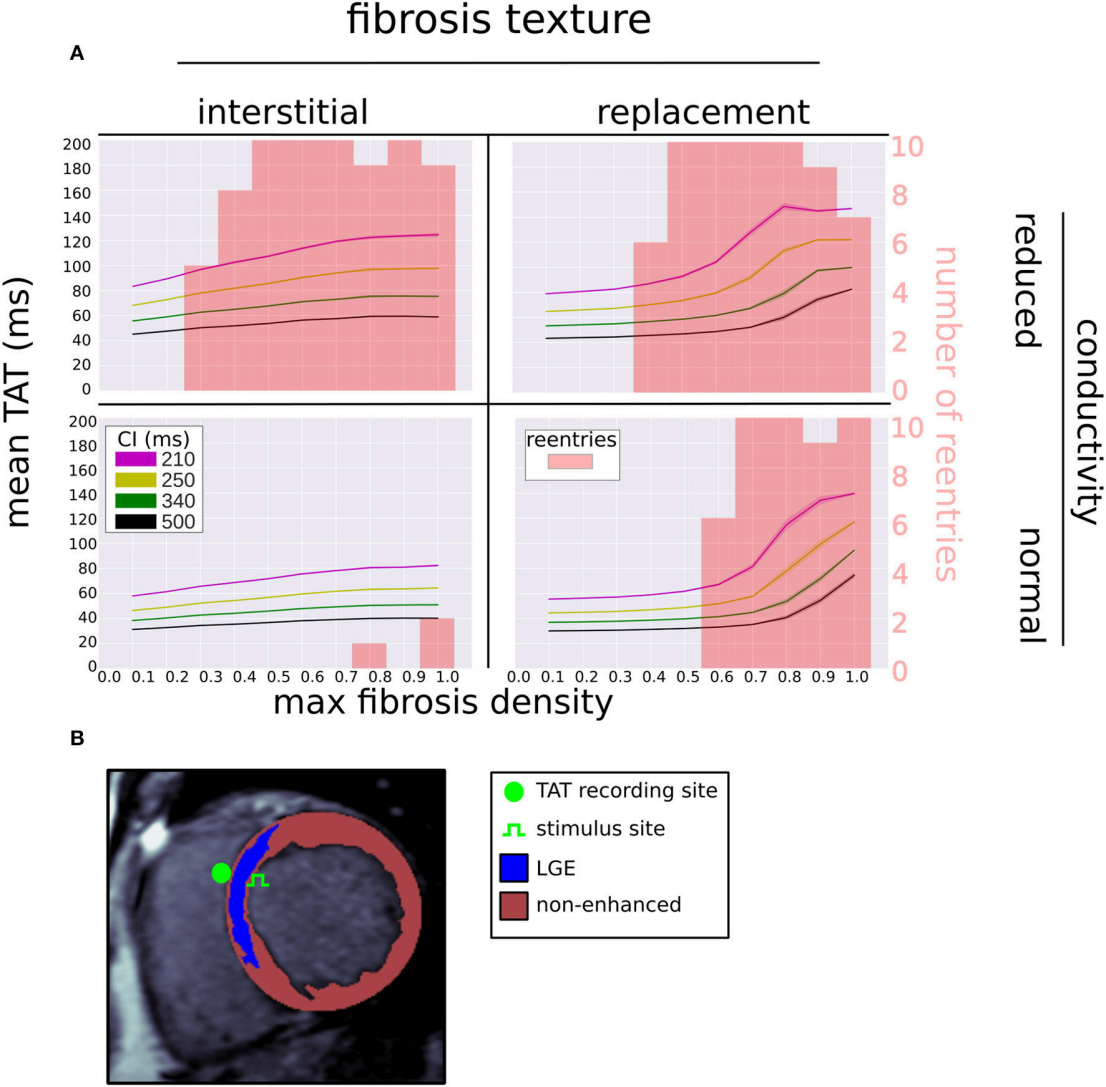
**(A)** Mean (solid line) and standard deviation (transparent area) of trans-septal activation time (TAT) for four coupling intervals (CI) vs. fibrosis density, fibrosis type, and LGE conductivity. The reentry incidence is overlaid in red. **(B)** Segmented LGE-CMR image with locations of stimulus and TAT recording site.

We examined the effects of fibrosis type and pacing rate on local activation patterns around the region of LGE. Zoomed in views of the activation maps at CIs, 500, 340, 250, 210 ms, for fibrosis densities 0.5, 0.8, and 1.0, along with the control case, are displayed in Figure 5. The activation maps show that both higher fibrosis density and faster pacing rate delayed activation. Furthermore, fibrosis density and pacing rate affected the overall pattern of activation, which fell into one of two types, direct or compartmentalized (Betensky et al., 2013). The direct pattern was characterized by circumferentially oriented isochrones, and activation times that reached their maximum on the anterior and posterior ends of the fibrosis region (Figure 5, control case). In contrast, compartmentalized activation was characterized by the presence of isochrones normal to the region of fibrosis, and a maximum activation time located directly across the pacing site on the right ventricular septum (Figure 5, replacement fibrosis, density 1.0). In some cases an increased pacing rate switched the activation pattern from direct to compartmentalized (Figure 5, replacement fibrosis, density 0.8), due to functional conduction block, a known precursor to reentry. We sampled the local activation time at a single point opposite the stimulus site, to obtain the trans-mural activation time (TAT). Our results in the top row of Figure 6 show that TAT always increased with faster pacing rate. Furthermore, TAT tended to increase with higher fibrosis density as well, especially with replacement fibrosis after the reentry threshold had been reached. This was due to large scale conduction block in the areas of fibrosis, which is confirmed by the correlation of the reentry incidence with zones of large TAT in Figure 6.

### 3.4. Transmural Activation Time With Varying Pacing Rate Predicts Reentry Inducibility

We tested the ability of TAT to predict reentry inducibility as a surrogate for knowledge of the fibrosis micro-structure. In particular we considered three metrics, TAT500, TAT210, and ΔTAT. TAT500 and TAT210 refer to TAT at CIs 500 and 210 ms, respectively, whereas ΔTAT refers to the difference between TAT210 and TAT500. Models for which reentry was inducible had significant higher mean values of the three metrics (Mann–Whitney *U*-test). For the inducible models these were ($\bar{\text{TAT500}}$ = 54.8 ms, $\bar{\text{TAT210}}$ = 115.3 ms, $\bar{\Delta \text{TAT}}$ = 61.1 ms) vs ($\bar{\text{TAT500}}$ = 38.9 ms, $\bar{\text{TAT210}}$ = 74.34 ms, $\bar{\Delta \text{TAT}}$ = 35.42 ms) for the non-inducible models.

**Figure 7:**
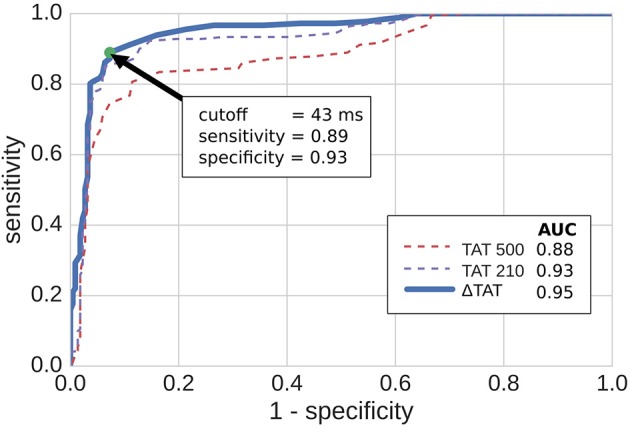
ROC curves for predicting reentry inducibility based on transmural activation times (TAT). TAT500 and TAT210 are the transmural activation times with 500 and 210 ms coupling intervals respectively, while ΔTAT is the difference. The green dot shows the point of maximum sensitivity and specificity. AUC stands for area under curve.

Using cut-off values of the TAT metrics, we predicted reentry inducibility using all of the model micro-structures. The results are summarized in Figure 7 as ROC curves, which show that all three TAT indicies are predictive of reentry. Furthermore, of the three indices, ΔTAT was the most predictive, and TAT500 the least (area under curve = 0.88, 0.92, 0.94 for TAT500, TAT210, ΔTAT respectively). This trend is explained by rate-dependant conduction block, occurring with a CI of 210 ms but not with a CI of 500 ms. As a consequence, TAT210 was a better predictor of reentry than TAT500. We designed ΔTAT to exploit rate-dependant conduction block even further. Such block gives a relatively small TAT500, a relatively large TAT210, and hence a large ΔTAT. Indeed this metric was the best reentry predictor, achieving a 89% sensitivity and 93% specificity at the point of maximum sensitivity and specificity (Youden Index). This compares favorably with the Youden Indicies of TAT500 (sensitivity 85%, specificity 94%) and TAT210 (sensitivity 81%, specificity 89%).

### 3.5. Reentry Incidence and Activation Delays Are Reduced at Off-Center Pacing Locations

**Figure 8:**
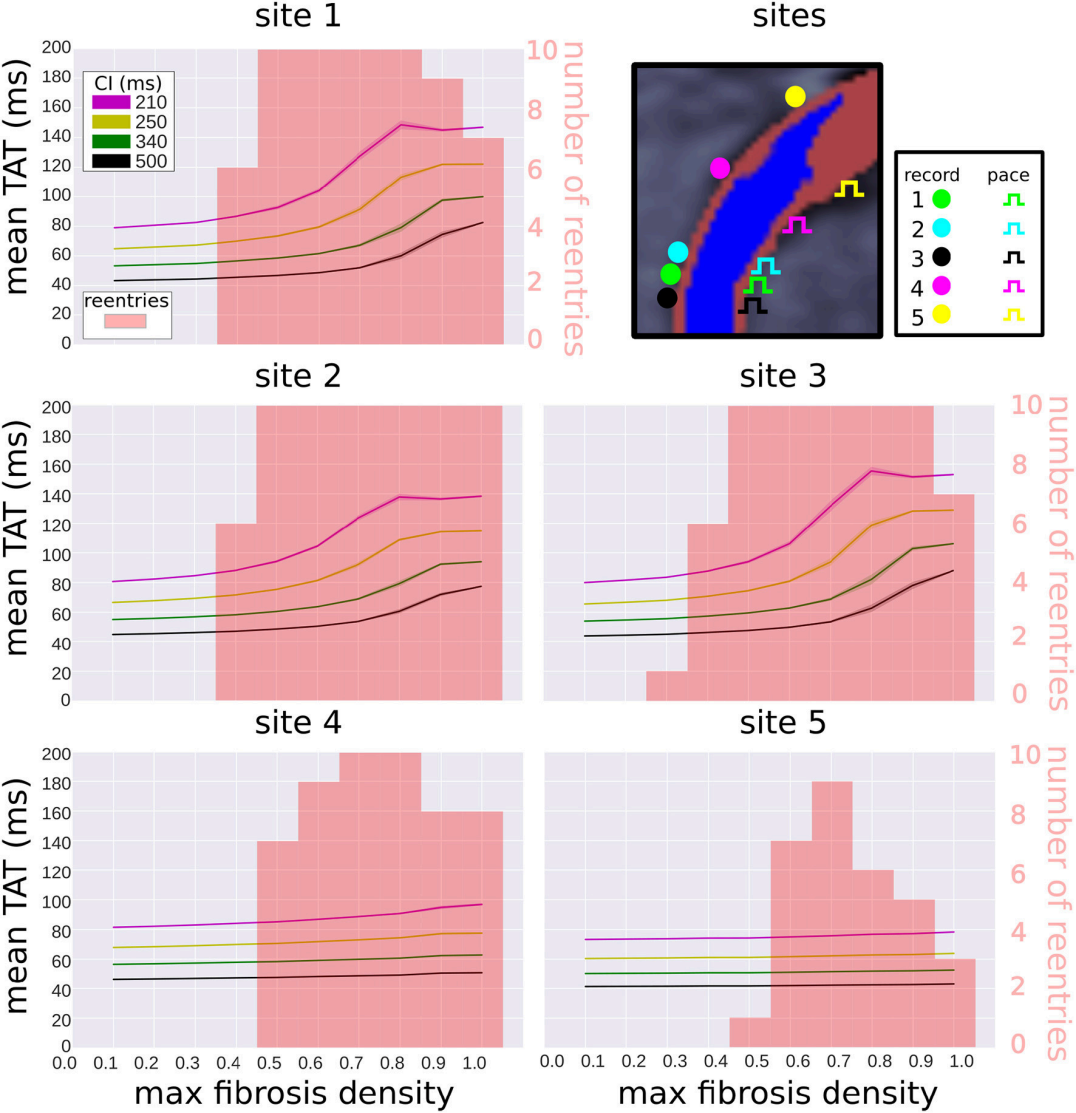
Effect of pacing location on transmural activation times and reentry incidence. Transmural activation times are given as a mean (solid line) and standard deviation (transparent area) for four coupling intervals (CI), taken over 10 random realizations of each fibrosis density. The reentry incidence is overlaid in red for comparison. All results were obtained with replacement fibrosis texture and reduced conductivity. The results for site 1 are the same as in Figure 6 (top right), whereas sites 2–5 are new locations shown for comparison.

We examined the effects of pacing and measuring TAT from alternate locations (see Figure 8 top right). Two of these locations (sites 2 and 3) are located very close to the original pacing location (site 1). Site 4 is located halfway between site 1 and the edge of the extent of the scar (site 5). The TAT times and reentry incidence for the alternative sites, with replacement fibrosis and reduced conductivity, are presented in Figure 8. For sites 2–3, the results are not very different from those obtained at site 1. At sites 4–5 the transmural activation times no longer correlate as much with the fibrosis density, and the reentry incidence is less. Site 5, which is furthest away from the center of the scar, has the lowest reentry incidence.

## 4. Discussion

Our study has highlighted the role of fibrosis density and local conduction slowing for the induction of reentry in NIDCM. Furthermore, we identified distinct reentry mechanisms related to the underlying fibrotic substrate, and found that variations in micro-structure density and conductivity had a measurable effect on activation patterns. This allowed for the accurate prediction of reentry inducibility in a wide variety of fibrotic micro-structures with pacing from the endocardium halfway through the extent of the scar.

### 4.1. A Model of Fibrosis for Non-ischemic Dilated Cardiomyopathy

To the best of our knowledge, our paper is the first to develop a computational model of fibrotic scarring in NIDCM. The main feature of our model is the incorporation of random fibrosis micro-structures. These micro-structures cannot be imaged with current clinical modalities, and were therefore approximated by our computational model.

Micro-structures have explained reentry formation in theoretical studies (Alonso and Bär, 2013), and in the context of atrial fibrillation (Roney et al., 2016; Vigmond et al., 2016), and have been shown to cause far-field effects when interacting with external electrical fields, such as those used for defibrillation (Fishler and Vepa, 1998). The densities of our fibrosis micro-structures were guided by LGE-CMR intensity values, which have been shown to reflect the relative volume fraction of fibrosis (Schelbert et al., 2010). A similar approach was also taken in representing fibrosis within the atria (Roney et al., 2016). However, the absolute fibrosis level for any particular non-ischemic LGE-CMR image is difficult to quantify (Glashan et al., 2018), which motivated our approach of systematically varying this parameter in our study.

We modeled reduced passive tissue conductivities within fibrotic areas, based on evidence of reduce gap junction protein expression in the context of fibrosis and dilated cardiomyopathy (Kostin et al., 2003; Glukhov et al., 2012), and showed that such effects greatly influence reentry formation in NIDCM. Similar results have been previously obtained in the context of ventricular infarcted areas (Arevalo et al., 2016) as well as the fibrotic atria (Roney et al., 2016). It is important to note, that any conduction slowing seen in our models is due to either: (a) RC (where present); or, (b) tortuous activation pathways through the micro-fibrosis patterns, which extend path lengths, and cause source-sink mis-matches which slow conduction due to increased electrotonic loading on the wavefront (De Bakker et al., 1993).

We modeled the excitability of fibrotic tissue in NIDCM as normal, which is supported by experimental studies (Anderson et al., 1993; Glukhov et al., 2012). This is in contrast to the majority of modeling representations of the infarcted border-zone, which often modulate sodium channel expression (Arevalo et al., 2016). Given the evidence of normal excitability, it is most likely that any ion channel changes in NIDCM would affect the later phases of the action potential and hence the action potential duration (APD). If any local APD effects were included in our NIDCM model, they would likely influence the reentry suspectibility (Clayton and Holden, 2005).

We did not include any models of myocyte-fibroblast coupling (MacCannell et al., 2007), which shorten action potential duration, and reduce excitability. The latter effect is not likely to be present in NIDCM, due to the normal excitability observed experimentally (Anderson et al., 1993; Glukhov et al., 2012). However, we expect that the action potential shortening effect, if included in our NIDCM model, would increase susceptibility to reentries, as was the case with atrial fibrosis (McDowell et al., 2013).

Finally, we did not include the effects of APD prolongation and altered transmural heterogeneity as were observed in Glukhov et al. (2012) for end-stage heart failure patients with NIDCM. This is because such effects are potentially a consequence of heart failure (Nattel et al., 2007). Hence their role in the acute phase of NIDCM is uncertain, which is when our computational model would be ideally used for prospective arrhythmic risk stratification. Further studies are needed to determine the extent to which APD prolongation and altered transmural heterogeneity play a role in acute NIDCM.

### 4.2. Mechanisms of Reentry

Previous studies have shown that fibrosis in NIDCM plays a role in both the initiation of block, and in the slowing of propagation (Anderson et al., 1993; Glukhov et al., 2012). Additionally, fibrosis has been associated with suppressed gap junction protein expression (Kostin et al., 2003; Glukhov et al., 2012), which reduces the conductivity of tissue, thereby contributing further to the slowing of conduction and formation of reentry.

By combining the known consequences of fibrosis in NIDCM into a computational model, we were able to simulate three reentry mechanisms, and analyse the relative contributions of fibrosis micro-structure and RC. We observed that macro-reentries involved large areas of functional and anatomical conduction block, which were more likely to form when the fibrosis was sufficiently dense, especially for replacement fibrosis with NC and interstitial fibrosis with RC.

We observed that rotor reentries formed when relatively large wavefronts pivoted and folded back onto an area of transient conduction block. The anisotropic properties of interstitial fibrosis facilitated this. Relatively free conduction in the transverse direction allowed for the preservation of large wavefronts in the fibrotic areas, whereas restricted conduction in the fiber direction caused zones of transient block which could be reentered by the pivoting waves. Indeed, most of the rotor reentries occurred with interstitial fibrosis. RC also played a key role in rotor formation, as only as single rotor was observed with NC.

We observed micro-reentries when propagation was constrained to a narrow pathway, and yet was not completely blocked. This occurred predominantly with replacement fibrosis and RC for the density range 0.6–0.9, at which micro-reentries were more common than macro-reentries. We did not observe micro-reentries with interstitial fibrosis. This was most likely due to the relatively unhindered lateral conduction of interstitial fibrosis which did not support the formation of narrow channels required for micro-reentry.

Previous simulation studies (Alonso and Bär, 2013; Vigmond et al., 2016), based on percolation theory, have also reported micro-reentry at distinct ranges of fibrosis density. These ranges were more narrow than what we observed, which we attribute to the heterogeneous density of our fibrosis micro-structures. This meant that different parts of our fibrotic zone could support micro-reentry, depending on the overall fibrosis density. For both micro-reentry and rotors, our results implicate the role of RC, as the incidence of these reentry types was much greater when the conductivity was reduced.

All reentries were initiated in the presence and absence of RC. The possibility that reentry in NIDCM is possible due to the structural effects of fibrosis alone, without RC, is also supported by previous simulation studies (Kazbanov et al., 2016). However, we show that the additional presence of RC increases the likelihood of reentry initiation. Similarly increased arrhythmic risks due to RC have been noted in the context of myocardial infarction (Cabo et al., 2006), and even in otherwise healthy hearts (Gutstein et al., 2001), which suggests that additional RC may also increase the risk of SCD in NIDCM.

### 4.3. Transmural Activation Patterns

A comparison of our simulated TAT values to literature provides a preliminary validation of our NIDCM fibrosis model. In particular, for our control case with no fibrosis and NC, we observed a TAT of 25 ms, which is within the range 26 ± 14 ms observed in healthy controls by Vassallo et al. (1986). For our models with fibrosis, we predicted TAT between 25–100 ms at the slow pacing rate. This is within the range 20–150 ms, measured previously in patients with septal scar (Betensky et al., 2013). The fact that we did not observe TAT values above 100 ms was most likely due to the intramural morphology of the scar in our particular patient. We expect that more transmural scars or thicker septal walls would produce higher TAT values.

We found that activation in the fibrotic region followed one of two patterns, direct or compartmentalized. These same patterns were observed by Betensky et al. (2013), with the compartmentalized pattern being attributed to greater scar volume and transmurality. Our simulations indicate the important role of scar density as well. Furthermore, we identified a particularly dangerous situation in which the activation pattern switched from direct to compartmentalized under an increase in pacing rate, reflecting a transient conduction block.

Our ROC analysis indicates that TATs are predictive of reentry. This is supported by Betensky et al. (2013), who reported that clinical VT were more common in patients with delayed TAT, and by Saumarez et al. (2003), who showed an association between SCD and rate dependant pacing delay. All three of our TAT metrics reflect this trend, with ΔTAT being particularly well suited to detecting large scale transient conduction block. Indeed, Figure 4 shows that, for replacement fibrosis, the difference in average TAT between CI 500 and 210 ms increased from about 90 ms in the reentry-free density regime to 100–200 ms for the densities that supported reentry. These reentries were overwhelmingly of the macro type, which involved large areas of conduction block that greatly increased ΔTAT values. For interstitial fibrosis, the link between ΔTAT and reentry was not as clear, most likely due to the presence of rotor reentries, which involved relatively small areas of conduction block and were therefore associated with modest ΔTAT values. Nevertheless, all three TAT metrics tended to increase with the interstitial fibrosis density, so that large TAT and ΔTAT values were indicative of being above the minimum density threshold for reentry.

### 4.4. The Effects of Pacing and TAT Recording Locations

We considered alternative pacing and TAT recording locations and measured the reentry incidence and TAT values at these sites. At the three sites in the center of the scar, the reentry incidence was similar and the TAT values correlated with scar density. At the off-center locations the reentry incidence was lower, with the site furthest away from the center of the scar experiencing the lowest incidence. This is expected since waves approaching the scar from the off-center locations traveled in a vector that was more greatly aligned with the myocardial fiber direction than for waves traveling from the central pacing locations. This meant that the effective CV of waves originating form the off-center locations was higher, and conduction block less likely.

Furthermore, TAT was only weakly correlated with the scar density at the off-center pacing sites. This was due to the effects of waves traveling around the scar, which were more likely to be the first to reach the TAT recording locations in the off-center setups. Since these waves traveled around the scar rather than through it, they experienced less fibrosis induced delay. For the pacing cite furthest away from the center of the scar, the scar transmurality was also substantially less, which lead to less delay for waves traveling through the scar than from the other pacing sites.

### 4.5. Clinical Implications

The DANISH trial (Køber et al., 2016) has demonstrated the need to improve the identification of patients with non-ischemic disease who benefit from ICD therapy. LGE-CMR, with its ability to image areas of fibrosis, is a promising tool for SCD risk stratification. Our results show that the type and density of the scar micro-structure underlying LGE play an important role in arrhythmia formation. This motivates the inclusion of data additional to LGE-CMR for the purposes of risk stratification. Our work suggests that electrical measurements, such as transmural activation times are an attractive possibility.

We demonstrated good performance of ΔTAT for predicting simulated reentry induciblity for a single scar macrostructure with a variety of miscrostructures. Further experiments with a variety of macrostructures are warranted. Our results with alternative pacing and TAT recording locations suggest that finding a location central to the scar is crucial for inferring the potential for reentry from TAT measurements. Furthermore, the method is robust to small changes in the pacing/recording locations. This is important for any potential clinical application, where less precision in determining locations can be expected.

Another potential data source complementary to LGE-CMR is T1 mapping, which allows for absolute comparisons to be made, and has been shown to be an independent predictor of arrhythmia (Chen et al., 2015). Future modeling efforts may benefit from the incorporation of both T1 and LGE-CMR.

Computational models of NIDCM, building on the methodology presented here, have the potential to predict SCD in a manner similar to that which has recently been demonstrated for ischemic disease (Arevalo et al., 2016). Future developments should address the *in-vivo* estimation of key fibrosis characteristics, such as density and fibrosis type, as well as the amount of conductivity reduction, as these would greatly improve the predictive capability of computational modeling, and thereby pave the way for personalized *in-silico* prediction of arrhythmic risk in NIDCM.

### 4.6. Limitations

All models were based on a single image. Consequentially our results do not account for geometric variability, such as wall thickness, scar transmurality, and scar extent. These factors may effect both TAT and reentry inducibility, and therefore the ability to infer reentry susceptibility from TAT measurements. Furthermore, our simulated TAT differences were a consequence of electrical restitution properties, which were governed by the Ten Tusscher and Panfilov (2006) model in our case. This model assumes normal ventricular restitution properties. Whether or not this is appropriate for fibrotic tissue in NIDCM is an open question. Additionally, we did not include any epi-endocardial differences in our cellular model, as the role of such differences in NIDCM is currently unknown.

As shown in section 3.5, the choice of pacing location is critical when making TAT measurements. In the clinical study of Betensky et al. (2013), this issue was handled by first surveying scars via voltage mapping and LGE-CMR, and then choosing an appropriate pacing location. We expect that similar techniques will be of use in future studies of scarring in NIDCM.

Simulations were performed in 2-D, which neglected the 3-D structure of ventricular tissue, myofibers, and interstitial clefts. As a consequence we restricted our study to the generation of reentry and not the sustenance, in which additional electrical pathways in 3-D may be important. Furthermore, micro-structural effects on TAT values could be less pronounced in 3-D at comparable fibrosis levels. This is because the presence of a 3rd dimension would likely increase the degree of connectivity among different paths through the scar, leading to quicker fill-in by multiple propagating activation wavefronts and hence more stable conduction. At the same time source-sink mismatches could be more pronounced in 3D due to the extra electrotonic load on a point in 3-D tissue. This could make conduction block and hence reentries more likely to occur.

Furthermore, the modeled 2-D fiber architecture did not account for the effects of transmural variation in fiber helix angle. Including this effect into a projection of the fibers onto a 2-D short-axis plane would cause the effective conductivity in the in-plane fiber direction to be less at the epicardial and endocardial edges. We expect that including this in our models would act to shorten the electrical wavelength around the scar, potentially increasing the reentry incidence.

Only two levels of conductivity reduction were tested: none and the heterogeneous scheme described in section 2.4. This was due to the lack of experimental data to inform the conductivity values, and to limit the number of model combinations.

Finally heterogeneous blocks of fibrosis corresponding to image pixels were present in our models, which have the potential to affect the probability of reentry (Kazbanov et al., 2016). However, our pixel size of 0.66 mm is well below the threshold of where block size effects were seen in Figure 2b of Kazbanov et al. (2016), which suggests that the fibrosis heterogeneity size was sufficiently resolved in our study.

## Author Contributions

All authors contributed to the interpretation of the data, drafting, and revising the manuscript, and approved the final version of the manuscript. The original study design was made by GB, MB, SP, BH, and discussed with the other authors. GB ran the simulations and analyzed the resulting data.

### Conflict of Interest Statement

The authors declare that the research was conducted in the absence of any commercial or financial relationships that could be construed as a potential conflict of interest.
